# Template synthesis of an intermediate in silver salt metathesis using a calix[4]arene-based diphosphine ligand†

**DOI:** 10.1039/d3dt00567d

**Published:** 2023-04-03

**Authors:** Jack Emerson-King, Adrian B. Chaplin

**Affiliations:** a Department of Chemistry, University of Warwick Gibbet Hill Road Coventry CV4 7AL UK a.b.chaplin@warwick.ac.uk

## Abstract

The synthesis and solid-state characterisation of the heterobimetallic rhodium(iii)/silver(i) complex [Rh(2,2′-biphenyl)(CxP_2_)Cl]⊃Ag^+^ is described, where CxP_2_ is a *trans*-spanning calix[4]arene-based diphosphine and the silver cation is datively bound to the chloride ligand within the cavity of the macrocycle.

## Abstract 2

Structural elucidation of Cl→Ag^+^ bonding using a cavitand-based ligand.

## Graphical abstract

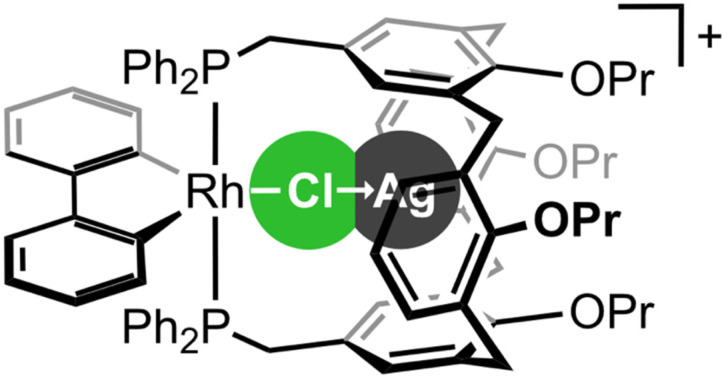

The activation of transition metal complexes by abstraction of halide ligands using silver(i) salts is a widely employed strategy in organometallic chemistry and catalysis.^1^ Mechanistic work by Mattson and Graham in 1981 substantiated a reaction sequence involving complexation of the silver(i) cation to the halide atom, before nucleophilic substitution and precipitation of the argentic salt from solution.^2^ Building on work by Reed and co-workers using weekly coordinating carborane anions,^3^ the first intermediate silver(i) halide adduct, [{CpMo(CO)_3_I}_2_(μ-Ag)_2_][CB_11_H_12_]_2_ was structurally corroborated in the solid-state by single crystal X-ray diffraction by Weller and co-workers in 2000.^4^ Notwithstanding facile onward reactivity, it is surprising to note that there have been only a handful of further well-defined examples over the intervening decades.^5^

As part of our group's ongoing interest in cavitand-based ditopic ligands,^6^ we have recently become engaged in exploring the coordination chemistry of Kubas’ calix[4]arene diphosphine ligand CxP_2_.^7^ In a preceeding paper we described the preparation of mononuclear rhodium(iii) aqua complex [Rh(biph)(CxP_2_)(OH_2_)][Al(OR^F^)_4_] (1-OH_2_; biph = 2,2′-biphenyl; R^F^ = C(CF_3_)_3_) by substitution of *trans*-[Rh(biph)(PPh_3_)_2_(OH_2_)][Al(OR^F^)_4_] with CxP_2_ in THF.^8^ Seeking to access water-free, low-coordinate Rh^III^(biph) derivative 1, preparation and subsequent silver(i)-based halide abstraction of 1-Cl was targeted. During the course of this work, we discovered that the silver(i) cation templates assembly of heterobimetallic rhodium(iii)/silver(i) complex [Rh(biph)(CxP_2_)Cl]⊃Ag^+^1-ClAg, which is a rare well-defined example of an intermediate in silver salt metathesis reactions.


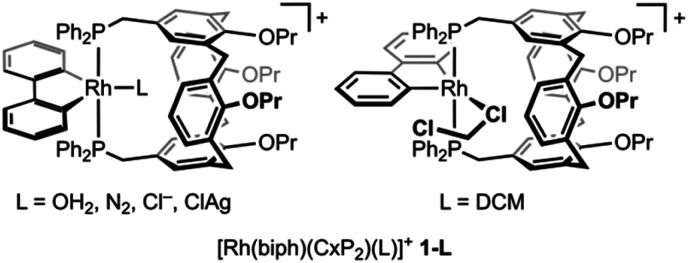
Monomeric rhodium(iii) complex [Rh(biph)(dtbpm)Cl] (dtbpm = bis(di-*tert*-butylphosphino)methane) is an effective source of the {Rh(biph)Cl} fragment in solution^9^ and was reacted with CxP_2_ in CH_2_Cl_2_ at RT. Substitution of dtbpm was observed alongside generation of a sparingly soluble product that exhibits a ^31^P resonance at *δ* 29.9 (^1^*J*_RhP_ = 114 Hz) and is assigned as dimeric [{Rh(biph)Cl}_2_(μ-CxP_2_)_2_] 2 on the basis of a low-quality X-ray structure determination (Fig. 1A). Whilst not the desired outcome, coordination of CxP_2_ in this manner is consistent with earlier reports.^7^

**Fig. 1:**
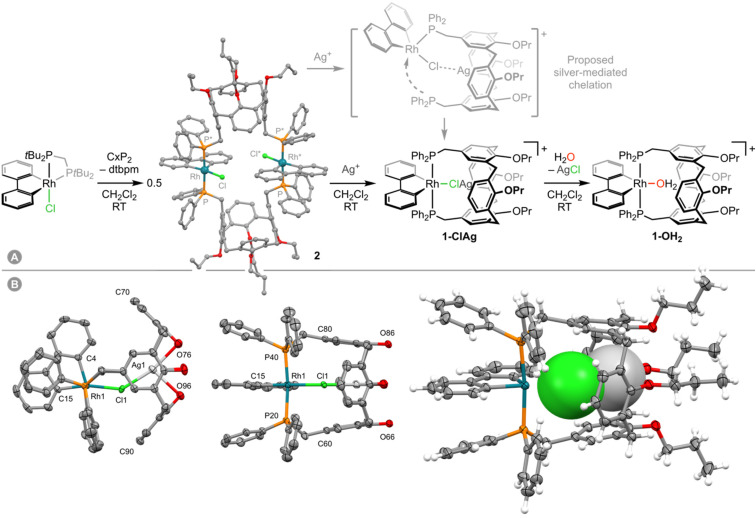
(A) Synthesis of 1-OH_2_ with ball and stick representation of one of the unique molecules of 2 in the solid-state (*Z*′ = 2) and H-atoms omitted for clarity; starred atoms are generated using the symmetry operation 2 − *x*,2 − *y*,1 − *z*. Reactions carried out under argon and [Al(OR^F^)_4_]^−^ counterions omitted. (B) Solid-state structure of 1-ClAg determined as a 58% : 42% mixture with 1-OH_2_ with thermal ellipsoids at 50% probability; solvent, and anion omitted. Two perspective views shown without H atoms and Pr groups, with a third showing the encapsulated ClAg unit in space fill with minor disordered components omitted (H_2_O, 2 × Pr). Selected bond lengths (Å) and angles (°): Rh1–Cl1, 2.403(2); C15–Rh1–Cl1, 166.68(12); Ag1–Cl1, 2.490(2); Ag1–O76, 2.484(3); Ag1–O96, 2.578(3); O76–Ag1–Cl1, 166.58(9); O76–Ag1–O96, 88.61(10); Rh1⋯Ag1 = 4.6271(6).

Reasoning that chelation of CxP_2_ could still be induced upon chloride abstraction, 2 was carried forward and reacted with two equivalents of Ag[Al(OR^F^)_4_] in dichloromethane under argon at RT. Analysis of the resulting suspension by NMR spectroscopy indicated clean conversion into a new complex within 48 h rather than the expected dichloromethane adduct 1-DCM (*δ*_31P_ 4.4, ^1^*J*_RhP_ = 117 Hz).^8^ This new organometallic is characterised by a sharp ^31^P resonance at *δ* 13.9 (^1^*J*_RhP_ = 120 Hz) significant downfield shifts of the aromatic ^1^H resonances of the calix[4]arene scaffold relative to 2 (*p*-Ar^H^, 6.02→7.32; *m*-Ar^H^, 5.63→7.11, *m*-Ar^P^, 6.22→6.50), and assigned to mononuclear 1-ClAg, where the CxP_2_ ligand adopts the desired *trans*-spanning coordination mode and the silver cation is bound within the cavity of the calix[4]arene scaffold (Fig. 1A). This species is persistent at RT under argon or dinitrogen, but incredibly moisture sensitive. Repeated attempts to isolate analytically pure samples were frustrated by facile and irreversible reaction with adventitious water,^10^ resulting in the formation of aqua complex 1-OH_2_ with concomitant precipitation of AgCl. Indeed, on a preparative scale, deliberate addition of a slight excess of water to *in situ* generated 1-ClAg enabled isolation of the considerably more robust, air and moisture stable 1-OH_2_ as an orange solid in 77% yield from 2. Consistent with the assigned structure of 1-ClAg, only a slight perturbation to the ^1^H and ^31^P resonances occurs on formation of 1-OH_2_ (*δ* 13.2, ^1^*J*_RhP_ = 120 Hz), alongside appearance of a distinctive 2H singlet at *δ* 0.84 for coordinated water.^8^ Most notably, one of the two unique OCH_2_ groups is shifted from 4.49→4.12 and we account for this change by coordination of the associated ether to silver in 1-ClAg.

Fortuitously, we have been able to structurally characterise 1-ClAg in the solid state through analysis of a co-crystalline sample formed with 1-OH_2_ (58% : 42% relative occupancy; Fig. 1B). From the crystallographic disorder model, silver was identified within the cavity and found to exhibit a pseudo T-shaped metal coordination geometry with a Ag1–Cl1 distance of 2.490(2) Å and two dative bonding interactions with the flanking ether units of the calix[4]arene (Ag1–O76, 2.484(3) Å; Ag1–O96, 2.578(3) Å). The latter presumably provides a decisive enthalpic driving force for formation of 1-ClAg.

Based on our observations, we propose conversion of 2 into 1-OH_2_ is initiated by capture of silver within the calix[4]arene scaffold. Chelation of CxP_2_ to rhodium is promoted by Cl→Ag^+^ bonding (Fig. 1A) and thereafter silver chloride is lost upon reaction with water, adventitious or deliberately added. This sequence further corroborates Mattson and Graham's mechanistic proposal for silver salt metathesis reactions, underscores the multifaceted ability of silver(i) cations to activate late transition metal complexes, and highlights the propensity of donor-functionalised cavitand ligands to orchestrate unusual metal-based reactivity.

## Conflicts of interest

The authors declare no conflicts of interest.

## Supplementary Material

DT-052-D3DT00567D-s001

DT-052-D3DT00567D-s002
