# Supplementary material for: Template synthesis of an intermediate in silver salt metathesis using a calix[4]arene-based diphosphine ligand
Source: Dalton Trans. 2023 Apr 3;52(16):5044–6. doi: 10.1039/d3dt00567d (PMC10127224; doi:10.1039/d3dt00567d)
Supplement: DT-052-D3DT00567D-s001 [file DT-052-D3DT00567D-s001.pdf]

Electronic Supplementary Material (ESI) for Dalton Transactions.
This journal is © The Royal Society of Chemistry 2023

Supporting information for:

Template synthesis of an intermediate in silver salt metathesis using a calix[4]arene-based diphosphine ligand

Jack Emerson-King and Adrian B. Chaplin.

Contents

1. General experimental methods. 2
2. Preparation [{Rh(biph)Cl}$_2$(*μ*-CxP$_2$)$_2$] **2** 2
3. NMR scale reaction between **2** and Ag[Al(OR^F)$_4$] 4
4. Attempted isolation of [Rh(biph)(CxP$_2$)(ClAg)][Al(OR^F)$_4$] **1-ClAg** 5
5. Preparation of [Rh(biph)(CxP$_2$)(OH$_2$)][Al(OR^F)$_4$] **1-OH$_2$** 7
6. References 8

1. General experimental methods.

All manipulations were performed under argon using standard Schlenk line and glove box techniques unless otherwise stated. Glassware was oven dried at 150 °C overnight and flamed under vacuum prior to use. 3 Å molecular sieves were activated by heating at 300 °C *in vacuo* overnight prior to use. Anhydrous solvents were obtained from commercial sources. Hexane was further dried over Na/K_2 alloy, vacuum-distilled, and freeze-pump-thaw degassed before being placed under argon over a potassium mirror. CH_2Cl_2 was further dried over CaH_2 overnight, vacuum-distilled, and freeze-pump-thaw degassed three times before being placed under argon over activated 3 Å molecular sieves. 1,2-Difluorobenzene (DFB) was stirred over neutral alumina, filtered, stirred over CaH_2 overnight, vacuum-distilled, and freeze–pump–thaw degassed three times before being placed under argon over activated 3 Å molecular sieves.[1] CD_2Cl_2 was placed over activated 3 Å molecular sieves and freeze-pump-thaw degassed three times before being placed under argon. [Rh(biph)(dtbpm)Cl][2] and CxP_2[3] were prepared using literature procedures, or minor variations thereof. For convenience, we have documented the full multi-step procedure for the latter in the supporting information of a preceding publication.[4] Ag[Al(OR^F)$_4$] was purchased from IoLiTec (https://iolitec.de/) and used as received. All other reagents and solvents are commercial products and were used as received. NMR spectra were recorded on Bruker spectrometers at 298 K unless otherwise stated. Chemical shifts are quoted in ppm and coupling constants in Hz. Virtual coupling constants are reported as the separation between the first and third lines.[5] In some instances, $^{31}P\{^1H\}$ NMR spectra were referenced using an internal sealed capillary of a 25 mM solution of trimethylphosphate in C_6D_6 (δ_{31P} 3.7).[6] Microanalyses were performed by Stephen Boyer at London Metropolitan University.

2. Preparation of [{Rh(biph)Cl}$_2$(μ-CxP_2)$_2$] 2

A solution of [Rh(biph)(dtbpm)Cl] (50.0 mg, 84.0 µmol) and CxP_2 (84.1 mg, 85.0 µmol) in CH_2Cl_2 (5 mL) was stirred at RT for 3 h. Excess Et_2O (*ca.* 45 mL) was added with stirring and the resulting precipitate isolated by filtration and dried *in vacuo*. Yield: 78.0 mg (30.5 µmol, 73%, amorphous yellow solid).

^{1}H NMR (CD_2Cl_2, 500 MHz): δ 7.33–7.28 (m, 4H, 6-biph), 7.31 (t, $^3J_{HH}$ = 7.4, 8H, *p*-Ph), 7.09 (t, $^3J_{HH}$ = 7.6, 16H, *m*-Ph), 7.03–6.95 (m, 16H, *o*-Ph), 6.80–6.76 (m, 8H, 3-biph+4-biph), 6.72–6.67 (m, 4H, 5-biph), 6.22 (s, 8H, *m*-Ar^P), 6.02 (t, $^3J_{HH}$ = 7.6, 4H, *p*-Ar^H), 5.63 (d, $^3J_{HH}$ = 7.6, 8H, *m*-Ar^H), 4.05 (d, $^2J_{HH}$ = 13.0, 8H, $ArCH_2Ar^P$), 3.78 (br t, $^3J_{HH}$ = 8.2, 8H, $Ar^POC\underline{H}_2$), 3.64 (br, 8H, CH_2P), 3.45 (br t, 8H, $^3J_{HH}$ = 6.9, $Ar^HOC\underline{H}_2$), 2.62 (d, $^2J_{HH}$ = 13.3, 8H $ArC\underline{H}_2Ar^P$), 1.81–1.68 (m, 16H, $C\underline{H}_2CH_3$), 0.99 (t, $^3J_{HH}$ = 7.4, 12H, $CH_2C\underline{H}_3$), 0.79 (t, $^3J_{HH}$ = 7.4, 12H, $CH_2C\underline{H}_3$).

$^{13}C\{^1H\}$ NMR (CD_2Cl_2, 126 MHz): δ 163.7 (dt, $^1J_{RhC}$ = 33, $^2J_{PC}$ = 8, 1-biph), 156.9 (s, *i*-Ar^P), 155.5 (s, *i*-Ar^H), 152.6 (s, 2-biph), 136.9 (s, *o*-Ar^P), 134.9 (vt, J_{PC} = 10, *o*-Ph), 133.33 (s, *o*-Ar^H), 133.25 (s, 6-biph), 131.0 (s, *m*-Ar^P), 130.1 (s, *p*-Ph), 129.0 (vt, J_{PC} = 44, *i*-Ph), 127.8 (vt, J_{PC} = 8, *m*-Ph), 127.7 (s, *m*-Ar^H), 127.7 (obscured, *p*-Ar^P), 125.0 (s, 5-biph), 123.1 (s, 4-biph), 122.1 (s, *p*-Ar^H), 121.7 (s, 3-biph), 77.5 (s, OCH_2), 76.7 (s, OCH_2), 31.0 (s, $Ar\underline{C}H_2Ar^P$), 30.1 (vt, J_{PC} = 20, CH_2P), 24.0 (s, $\underline{C}H_2CH_3$), 23.2 (s, $\underline{C}H_2CH_3$), 11.1 (s, $CH_2\underline{C}H_3$), 10.1 (s, $CH_2\underline{C}H_3$).

$^{31}P\{^1H\}$ NMR (CD_2Cl_2, 162 MHz): δ 29.9 (d, $^1J_{RhP}$ = 114).

Anal. Calcd for $C_{156}H_{156}Cl_2O_8P_4Rh_2$ (2559.76 g·mol^{-1}): C, 73.20; H, 6.14; N, 0.00. Found: C, 73.12; H, 5.99; N, 0.00.

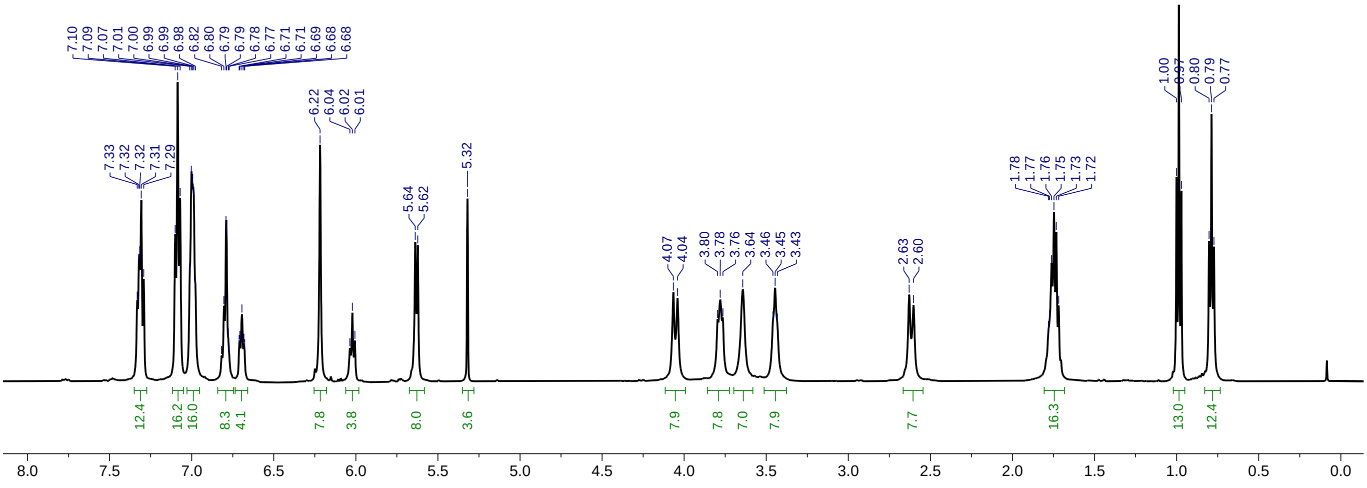

Figure S1. ^1H NMR spectrum of **2** (CD_2Cl_2, 500 MHz).

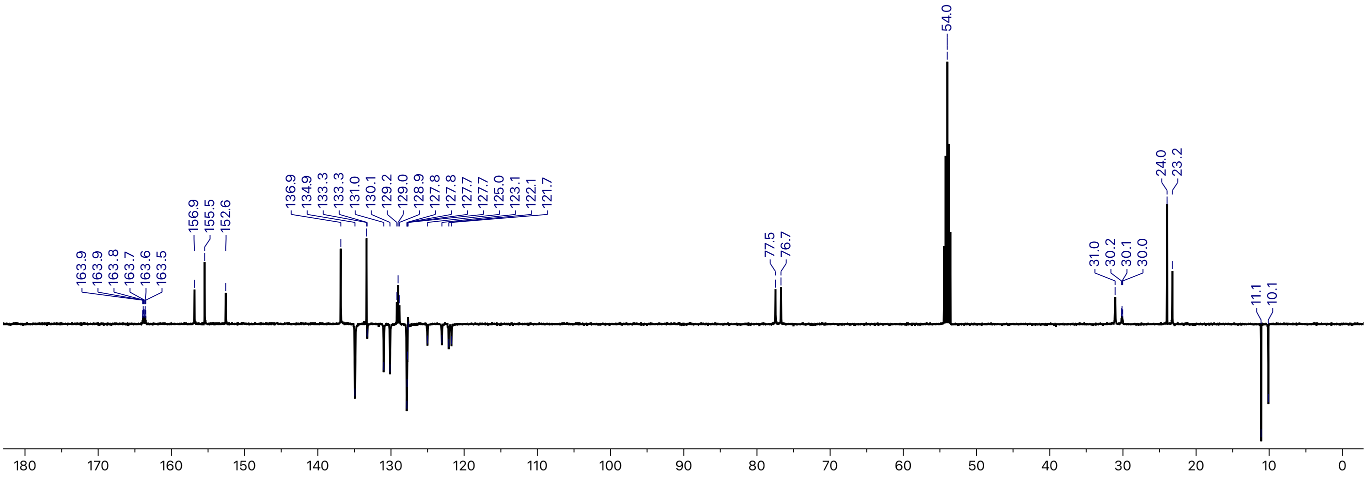

Figure S2. $^{13}C\{^1H\}$ APT NMR spectrum of **2** (CD_2Cl_2, 126 MHz).

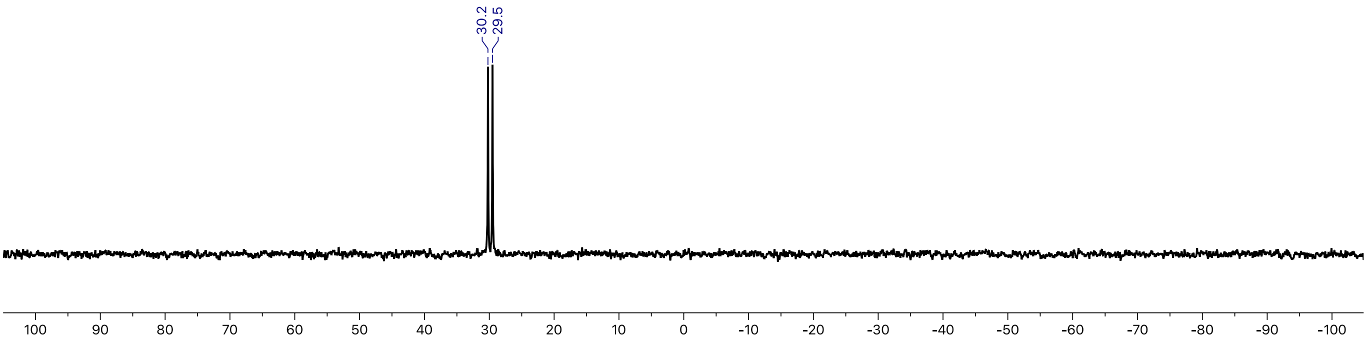

Figure S3. $^{31}P\{^1H\}$ NMR spectrum of **2** (CD_2Cl_2, 162 MHz).

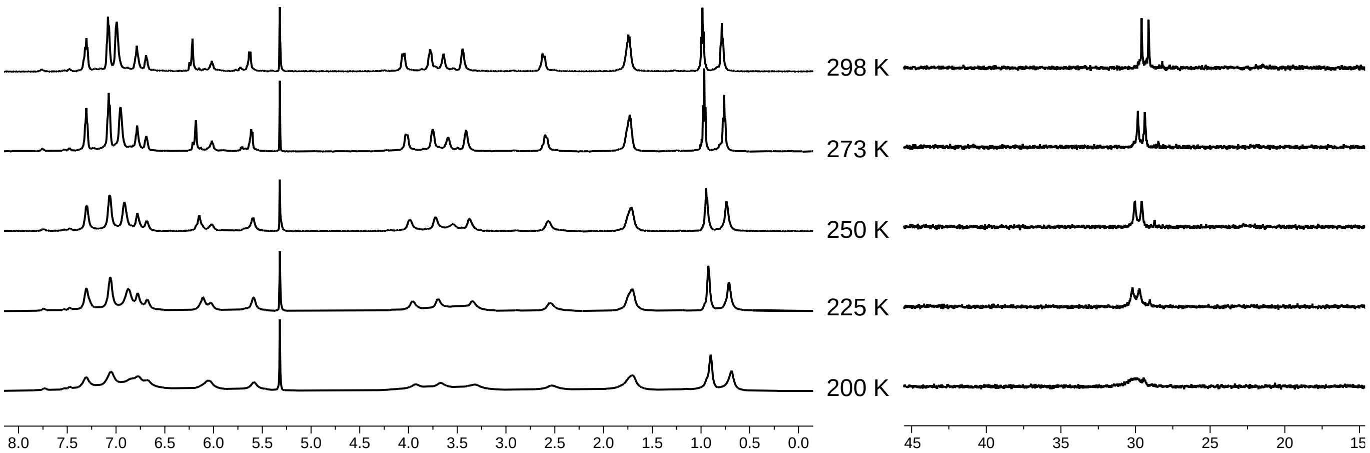

Figure S4. Variable temperature ^{1}H and $^{31}P\{^{1}H\}$ NMR spectra of **2** (CD_2Cl_2, 600/243 MHz).

3. NMR scale reaction between 2 and Ag[Al(OR^F)$_4$]

A suspension of [{Rh(biph)Cl}$_2$(μ-CxP$_2$)$_2$] (6.4 mg, 2.5 µmol) and Ag[Al(OR^F)$_4$] (5.4 mg, 5 µmol) in CD_2Cl_2 (0.5 mL) was agitated within a J. Young's valve NMR tube at RT. Analysis by NMR spectroscopy indicated complete conversion into [Rh(biph)(CxP$_2$)(ClAg)][Al(OR^F)$_4$] within 48 h.

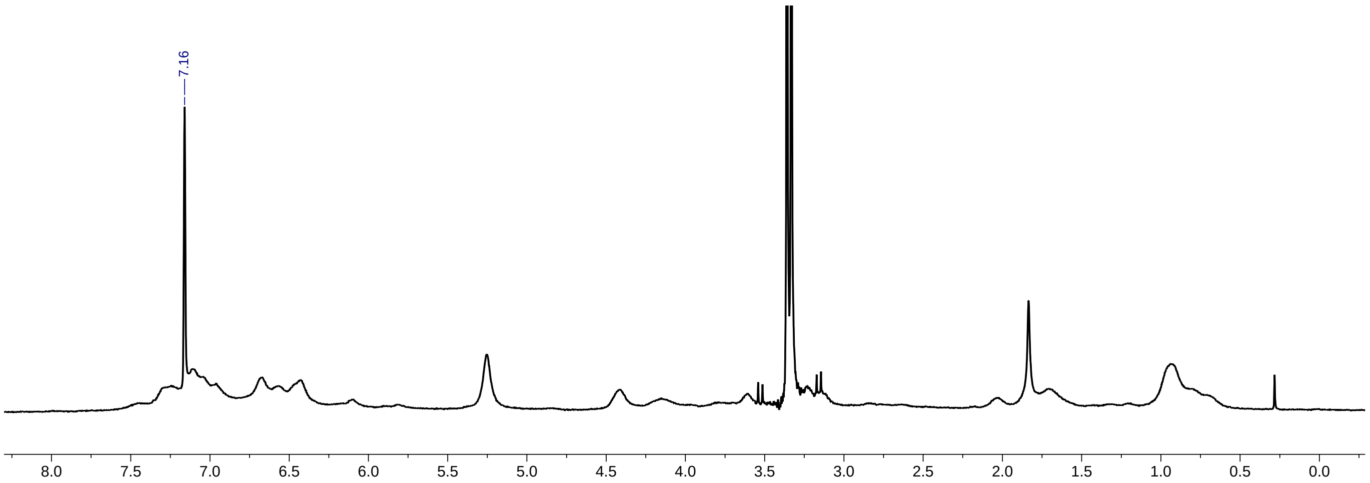

Figure S5. ^{1}H NMR spectrum collected during the reaction between **2** and Ag[Al(OR^F)$_4$] with internal O=P(OMe)$_3$ standard (CD_2Cl_2, 400 MHz).

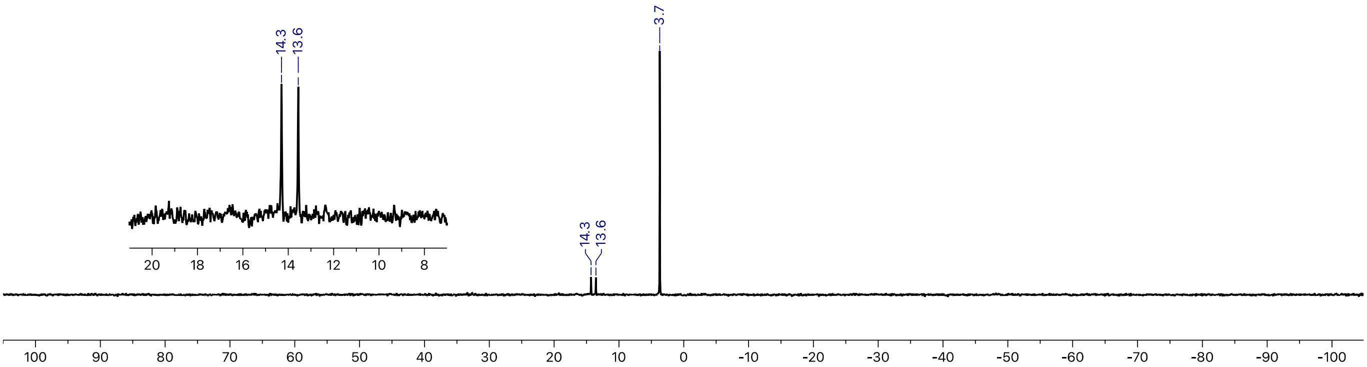

Figure S6. $^{31}P\{^{1}H\}$ NMR spectrum collected during the reaction between **2** and $Ag[Al(OR^F)_4]$ with internal $O{=}P(OMe)_3$ standard (CD_2Cl_2, 162 MHz).

4. Attempted isolation of $[Rh(biph)(CxP_2)(ClAg)][Al(OR^F)_4]$ 1-ClAg

A suspension of $[\{Rh(biph)Cl\}_2(\mu\text{-}CxP_2)_2]$ (20 mg, 7.8 µmol) and $Ag[Al(OR^F)_4]$ (16.8 mg, 15.6 µmol) in CH_2Cl_2 (5 mL) was stirred at RT for 48 h. The solution was filtered through a dried glass microfibre filter and volatiles removed in vacuo. The residue was extracted into CD_2Cl_2 to afford the product in *ca.* 85% purity which characterised *in situ* by NMR spectroscopy. Attempted purification by recrystallisation from CH_2Cl_2/hexane lead to complete conversion into $[Rh(biph)(CxP_2)(OH_2)][Al(OR^F)_4]$. On one occasion, when the reaction was carried out in DFB, we were able to obtain a sample of $[Rh(biph)(CxP_2)(L)][Al(OR^F)_4]$ (L = AgCl, H_2O) suitable for X-ray diffraction following filtration and diffusion of hexane at RT.

^{1}H NMR (CD_2Cl_2, 400 MHz, selected data): δ 7.38 (t, $^{3}J_{HH}$ = 7.3, 4H, *p*-Ph), 7.32 (t, $^{3}J_{HH}$ = 7.5, 4H, *p*-Ar^H), 7.18 (t, $^{3}J_{HH}$ = 7.3, 8H, *m*-Ph), 7.11 (d, $^{3}J_{HH}$ = 7.5, 8H, *m*-Ar^H), 6.79–6.71 (m, 8H, *o*-Ph), 6.50 (s, 4H, *m*-Ar^P), 4.51 (d, $^{2}J_{HH}$ = 13.1, 4H, $ArCH_2Ar^P$), 4.52–4.43 (m, 4H, $ArOC\underline{H}_2$), 3.68 (t, $^{3}J_{HH}$ = 7.3, 4H, $ArOC\underline{H}_2$), 3.30 (br, 4H, CH_2P), 3.21 (d, $^{2}J_{HH}$ = 13.1, 4H, $ArCH_2Ar^P$), 2.18 – 2.03 (m, 4H, $C\underline{H}_2CH_3$), 1.92 (app sex, $^{3}J_{HH}$ = 7.4, 4H, $C\underline{H}_2CH_3$), 1.04 (t, $^{3}J_{HH}$ = 7.5, 6H, $CH_2C\underline{H}_3$), 0.99 (t, $^{3}J_{HH}$ = 7.3, 6H, $CH_2C\underline{H}_3$),

$^{31}P\{^{1}H\}$ NMR (CD_2Cl_2, 162 MHz): δ 14.0 (d, $^{1}J_{RhP}$ = 120).

$^{19}F\{^{1}H\}$ NMR (CD_2Cl_2, 377 MHz): δ -75.7 (s).

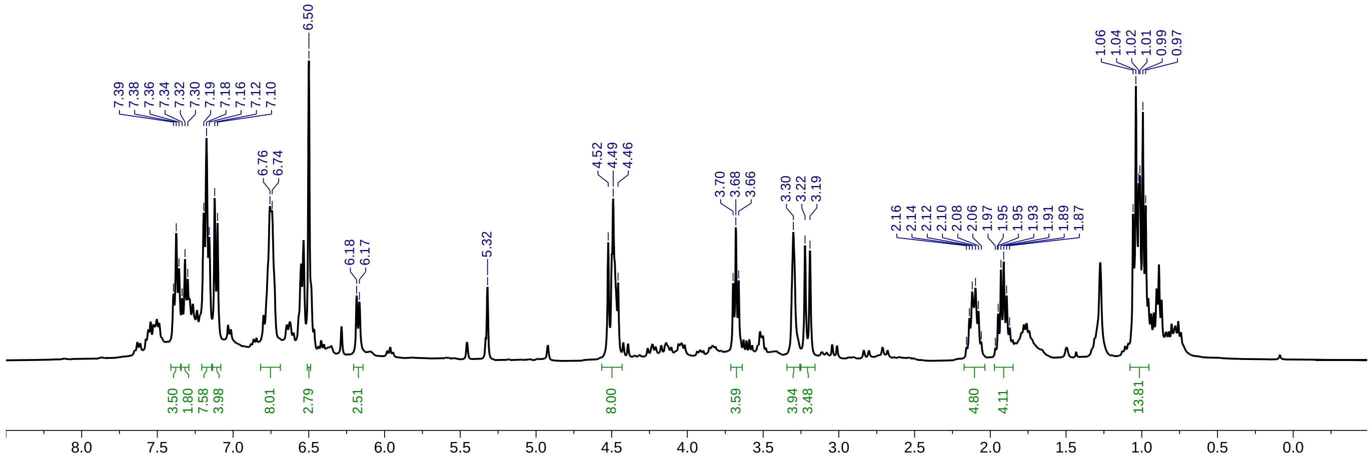

Figure S7. ^{1}H NMR spectrum of **1-ClAg** (CD_2Cl_2, 400 MHz).

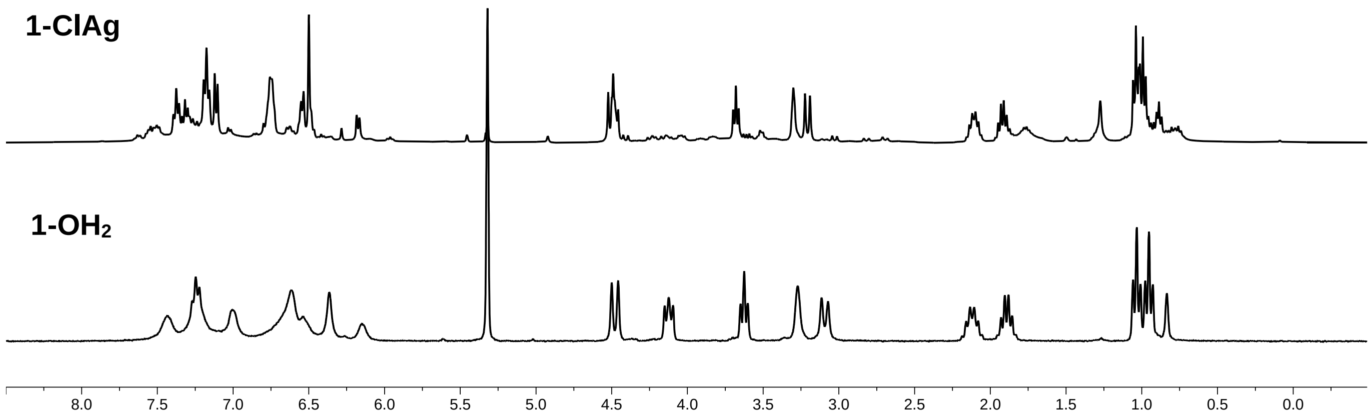

Figure S8. ^{1}H NMR spectra of **1-ClAg** and **1-OH₂** (CD_2Cl_2, 400 MHz).

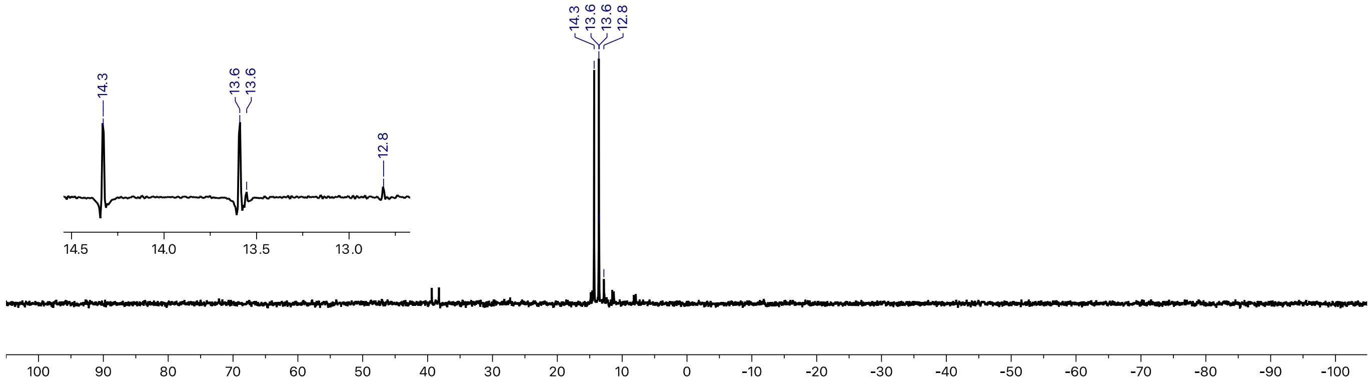

Figure S9. ^{31}P{^{1}H} NMR spectrum of **1-ClAg** (CD_2Cl_2, 162 MHz). Insert with sine bell and without exponential apodization to show presence of **1-OH₂**.

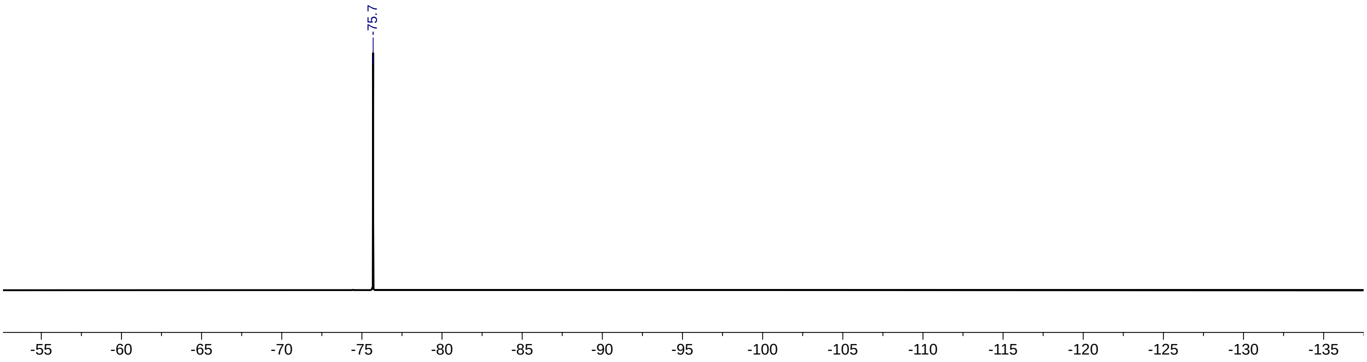

Figure S10. $^{19}F\{^1H\}$ NMR spectrum of **1-ClAg** (CD_2Cl_2, 377 MHz).

5. Preparation of $[Rh(biph)(CxP_2)(OH_2)][Al(OR^F)_4]$ 1-OH₂

A suspension of $[\{Rh(biph)Cl\}_2(\mu\text{-}CxP_2)_2]$ (128.8 mg, 50 µmol) and $Ag[Al(OR^F)_4]$ (107.5 mg, 100 µmol) in CH_2Cl_2 (10 mL) was vigorously stirred at RT for 18 h. H_2O (2.7 µL, 150 µmol) was added, precipitating AgCl, and the yellow solution filtered in air. The solvent was concentrated *in vacuo* to *ca.* 5 mL and layered with wet hexane (*ca.* 45 mL) in air to afford the product along with further AgCl precipitate. The product was extracted into CH_2Cl_2 (*ca.* 5 mL) and the recrystallisation procedure repeated until no further AgCl precipitate was observed. Yield: 171 mg (76.7 µmol, 77%, orange crystalline blocks). Spectroscopic data are consistent with the literature.[4]

$^{31}P\{^1H\}$ NMR (CD_2Cl_2, 162 MHz): δ 13.2 (d, $^1J_{RhP}$ = 120).

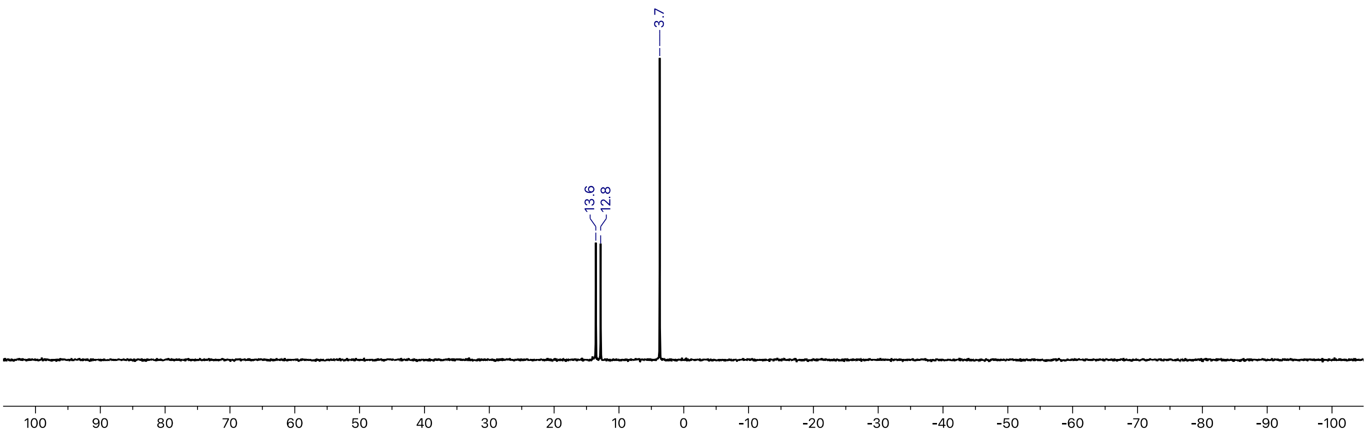

Figure S11. $^{31}P\{^1H\}$ NMR spectrum of **1-OH₂** with internal $O{=}P(OMe)_3$ standard (CD_2Cl_2, 162 MHz).

6. References

[1] S. D. Pike, M. R. Crimmin and A. B. Chaplin, *Chem. Commun.*, 2017, **53**, 3615–3633.

[2] C. N. Iverson and W. D. Jones, *Organometallics*, 2001, **20**, 5745–5750.

[3] X. Fang, B. L. Scott, J. G. Watkin, C. A. G. Carter and G. J. Kubas, *Inorg. Chim. Acta.*, 2001, **317**, 276–281.

[4] J. Emerson-King, S. Pan, M. R. Gyton, R. Tonner-Zech and A. B. Chaplin. *Chem. Commun.*, 2023, **59**, 2150–2152.

[5] P. S. Pregosin, *NMR in Organometallic Chemistry*, Wiley-VCH, 2012, pp. 251–254.

[6] R. Streck and A. J. Barnes, *Spectrochim. Acta A*, 1999, **55**, 1049–1057.
